# Charcot–Marie–Tooth-like presentation in giant axonal neuropathy: clinical variability and prevalence in a large Japanese case series

**DOI:** 10.1007/s00415-025-13243-5

**Published:** 2025-07-16

**Authors:** Takahiro Hobara, Masahiro Ando, Yujiro Higuchi, Jun-Hui Yuan, Akiko Yoshimura, Takashi Saito, Takashi Shiihara, Shiho Okuda, Naoki Fukushima, Hiroyuki Awano, Takahito Inoue, Chikashi Yano, Fumikazu Kojima, Kento Kodama, Yu Hiramatsu, Satoshi Nozuma, Tomonori Nakamura, Yusuke Sakiyama, Akihiro Hashiguchi, Jun Mitsui, Shoji Tsuji, Hiroshi Takashima

**Affiliations:** 1 Department of Neurology and Geriatrics, Kagoshima University Graduate School of Medical and Dental Sciences, 8-35-1 Sakuragaoka, Kagoshima City, Kagoshima, 890-8520 Japan; 2 Department of Child Neurology, National Center Hospital, National Center of Neurology and Psychiatry, Tokyo, Japan; 3 Department of Neurology, Gunma Children’s Medical Center, Gunma, Japan; 4 Department of Neurology, Hyogo Prefectural Kakogawa Medical Center, Hyogo, Japan; 5 Department of Pediatrics, Nakatsu Municipal Hospital, Oita, Japan; 6 Research Initiative Center, Organization for Research Initiative and Promotion, Tottori University, Tottori, Japan; 7 Center for Maternal, Fetal and Neonatal Medicine, Fukuoka University Hospital, Fukuoka, Japan; 8 Department of Neurology, National Hospital Organization Okinawa Hospital, Okinawa, Japan; 9 Department of Precision Medicine Neurology, Graduate School of Medicine, The University of Tokyo, Tokyo, Japan; 10 Institute of Medical Genomics, International University of Health and Welfare, Chiba, Japan; 11 Department of Neurology, The University of Tokyo Hospital, Tokyo, Japan

**Keywords:** Giant axonal neuropathy, Gigaxonin, Inherited peripheral neuropathy, Charcot–Marie–Tooth disease, Next-generation sequencing, Phenotypic heterogeneity

## Abstract

**Background:**

Giant axonal neuropathy 1 (GAN) is a rare neurodegenerative disorder with autosomal recessive inheritance and significant phenotypic heterogeneity, ranging from milder presentations resembling Charcot–Marie–Tooth disease (CMT) to classical presentations involving central and peripheral nervous systems. We investigated the genetic and clinical spectrum of GAN in Japanese patients with inherited peripheral neuropathies (IPNs).

**Methods:**

We conducted genetic screening of 3315 Japanese patients diagnosed with IPNs between 2007 and 2023 using targeted next-generation or whole-exome sequencing. Variant pathogenicity, clinical features, and neurophysiological and neuroimaging findings were reviewed.

**Results:**

We identified seven biallelic *GAN* variants in five patients from four unrelated families, including one homozygous and three compound heterozygous genotypes. Two novel pathogenic variants were identified: c.922G > T (p.Glu308*) and c.456dup (p.Ala153Cysfs*27). Two families exhibited the classical phenotype, whereas the other two exhibited a CMT-like phenotype. Mean onset age was 4.4 years (range 1.5–8), and gait disturbance was the initial symptom. The most common findings included distal weakness (n = 5), sensory impairment (n = 4), scoliosis (n = 3), autonomic dysfunction (n = 2). Neurophysiologically, all patients had sensorimotor axonal polyneuropathy. One patient with mild phenotype maintained a CMT-like state without systemic involvement until the age of 43 years and was still alive at 72, representing the longest documented survival in GAN.

**Conclusion:**

This study expands the genetic and phenotypic spectrum of GAN by identifying novel variants and a long-term survivor. These findings underscore the importance of systematic genetic screening for *GAN* in pediatric-onset CMT, even in the absence of classical features.

**Supplementary Information:**

The online version contains supplementary material available at 10.1007/s00415-025-13243-5.

## Introduction

Giant axonal neuropathy 1 (GAN, OMIM: #256850) is a rare autosomal recessive genetic disorder and progressive neurodegenerative condition characterized by an early age of onset and severe symptoms. Asbury et al. first described the clinical and pathological features of GAN in 1972 [1]. The disease is associated with mutations in *GAN* (NM_022041.4, GRCh38) encoding gigaxonin, wherein biallelic variants resulting in loss of function of gigaxonin lead to intermediate filament dysfunction, ultimately causing neurodegeneration [2, 3]. In classical presentation of GAN, individuals typically exhibit central nervous system (CNS) involvement–such as cranial nerve involvement, bulbar palsy, cerebellar signs, and intellectual disability–kinky hair, and peripheral nervous system involvement characterized by axonal peripheral neuropathy manifesting before the age of 3, and their life expectancy is generally limited to the third decade of life [4–6]. Conversely, late-onset presentations predominantly feature axonal peripheral neuropathy with minimal to no CNS involvement [4, 5, 7, 8]. These milder cases of GAN are classified as type 2 Charcot–Marie–Tooth disease (CMT).

CMT is a genetically heterogeneous and difficult-to-treat disorder with > 140 causative genes [9]. However, *GAN* is among the few CMT-associated genes for which a therapeutic approach is currently under investigation [10, 11]. A Phase 1 clinical trial involving the intrathecal administration of an adeno-associated viral (AAV) vector-based gene therapy reported promising results, raising expectations for further therapeutic advancements [12]. Identifying *GAN* mutations in patients clinically suspected for CMT could have significant clinical benefits; particularly, a definitive diagnosis of GAN could open avenues for treatment.

Herein, we analyzed a large case series of Japanese individuals clinically diagnosed with inherited peripheral neuropathies (IPNs) (n = 3315) and performed *GAN* analysis to identify affected patients, characterize the clinical features, and broaden the known phenotypic spectrum of GAN.

## Materials and methods

### Sample selection

We enrolled 3315 unrelated Japanese patients clinically diagnosed with IPNs/CMT, through a nationwide genetic study conducted between 2007 and 2023, including 865 patients with disease onset before 10 years of age. All patients were examined and diagnosed by their respective neurologists, and their clinical data and blood samples were transferred to our laboratory for genetic testing. All patients with demyelinating type were confirmed to be negative for *PMP22* duplication/deletion using fluorescence in situ hybridization or multiplex ligation-dependent probe amplification.

### Microarray analysis, next-generation sequencing, and whole-exome sequencing

For all analyses, genomic DNA was extracted from peripheral blood using a Puregene Core Kit C (QIAGEN, Hilden, Germany) following the manufacturer’s instructions. Between 2007 and 2012, we performed genetic screening in 417 patients using DNA microarray (Affymetrix, Inc., Santa Clara, CA, USA) targeting disease-causing or candidate gene panels for IPNs; however, this panel did not include *GAN*. Additionally, whole-exome sequencing was performed using the HiSeq2000/HiSeq2500 platform (Illumina Inc., San Diego, CA, USA) or Ion Proton system (Thermo Fisher Scientific, Waltham, MA, USA) in 273 patients who tested negative for pathogenic variants on DNA microarray. Since 2012, we have conducted genetic screening of 2898 patients using an in-house gene panel, targeting disease-causing genes that includes *GAN*. The screening was performed via next-generation sequencing using Illumina MiSeq (Illumina) or Ion Proton platform. The general workflow is presented in Supplementary Fig. 1.

### Data analysis and variant interpretation

To interpret the pathogenic significance of all identified *GAN* variants, variants were checked against public control databases (Genome Aggregation Database [gnomAD; https://gnomad.broadinstitute.org] [13] and Japanese Multi Omics Reference Panel [jMorp; https://jmorp.megabank.tohoku.ac.jp] [14]) and our in-house database. Additionally, multiple in silico tools were applied to predict the potential impact of the identified variants, including SIFT4G (https://sift.bii.a-star.edu.sg/sift4g/AboutSIFT4G) [15], PolyPhen2 (http://genetics.bwh.harvard.edu/pph2) [16], MutationTaster (http://www.mutationtaster.org) [17], FATHMM (http://fathmm.biocompute.org.uk) [18], CADD (https://cadd.gs.washington.edu) [19], and BayesDel (https://fenglab.chpc.utah.edu/BayesDel) [20]. We utilized MetaDome (https://stuart.radboudumc.nl/metadome) [21] to assess the overall tolerance score of gigaxonin. All variants were validated using Sanger sequencing.

The detected novel variants were evaluated according to the guidelines of the American College of Medical Genetics and Genomics and the Association for Molecular Pathology (ACMG–AMP) and the ClinGen Sequence Variant Interpretation (https://clinicalgenome.org/working-groups/sequence-variant-interpretation/) [22–26].

## Results

### Genetic findings

**Fig. 1:**
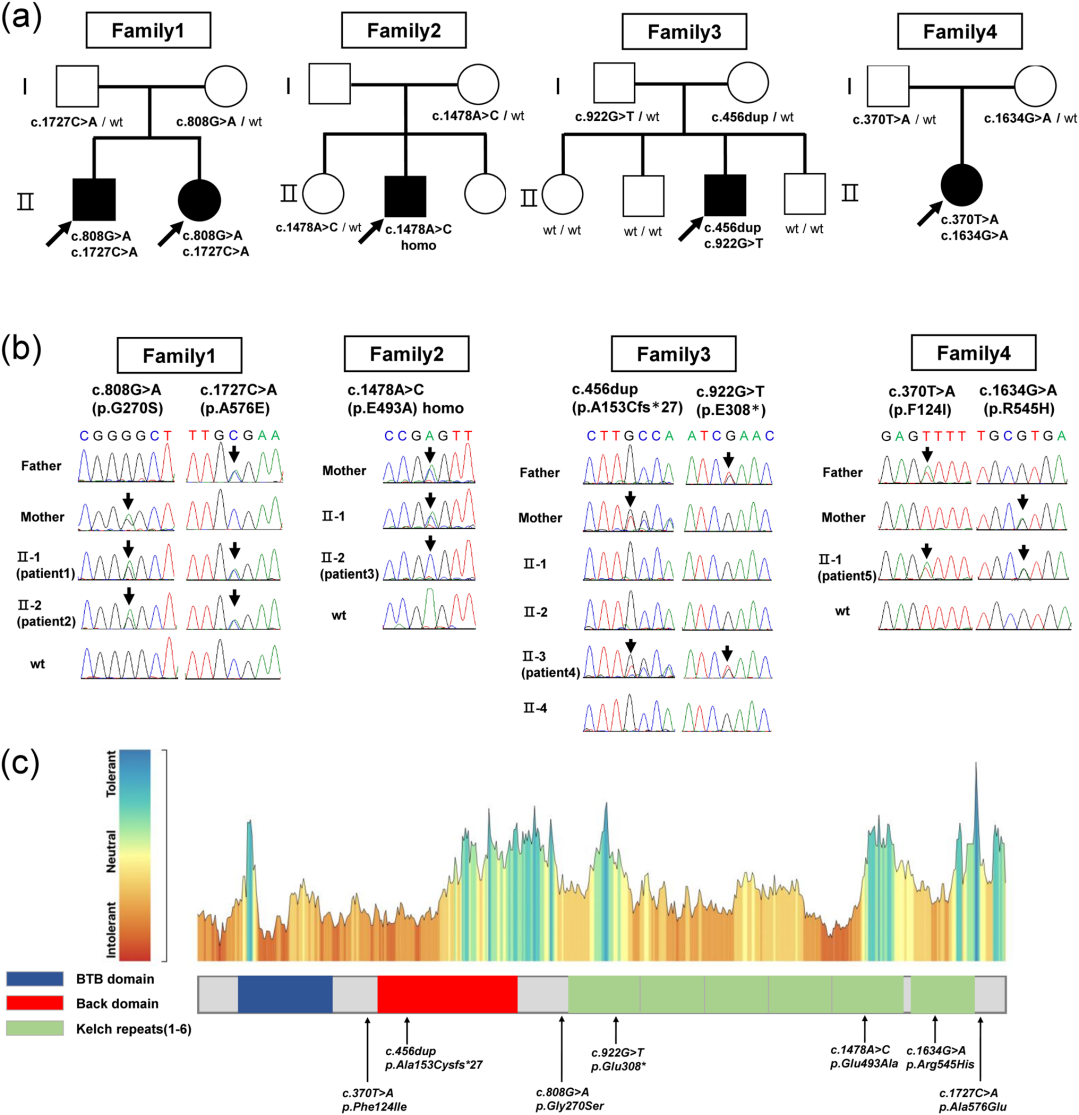
Analysis of *GAN* variants, pedigree analysis, and conservation analysis in four families. **A** Biallelic variants in the GAN gene were identified in five individuals across four families. Families 1, 3, and 4 exhibited compound heterozygous variants, whereas Family 2 had a homozygous variant. Arrows indicate probands. wt, wild type. **B** Sanger sequencing of the GAN gene in each family. Arrows indicate the locations of gene mutations. In Families 1, 3 and 4, the parents were heterozygous for the mutations, with affected individuals having biallelic mutations and unaffected individuals being either heterozygous or wild type, indicating segregation. In Family 2, the affected individual had a homozygous mutation, whereas the mother and unaffected sibling were heterozygous. Genetic analysis could not be conducted in the father. **C** The Schematic diagram of the GAN domains and the tolerance landscape of the GAN protein from MetaDome. Although the gene mutations identified in the four families in this study span various regions, they are predominantly located within intolerant regions

We identified seven *GAN* (NM_022041.4, GRCh38) variants in five patients from four unrelated families among the 3315 Japanese patients with IPNs (0.12%). The detected variants included the compound heterozygous variants c.808G > A (p.Gly270Ser) and c.1727C > A (p.Ala576Glu) in Family 1, the homozygous variant c.1478A > C (p.Glu493Ala) in Family 2, the compound heterozygous variants c.456dup (p.Ala153Cysfs*27) and c.922G > T (p.Glu308*) in Family 3, and the compound heterozygous variants c.370 T > A (p.Phe124Ile) and c.1634G > A (p.Arg545His) in Family 4 (Fig. 1A, B).

Among these variants, one frameshift variant was located in the broad-complex, tramtrack, bric-à-brac and C‑terminal Kelch (BACK) domain, one nonsense variant and two missense variants were located in the Kelch domain, and the remaining three missense mutations were located in linker regions with unknown functional role (Fig. 1C). Conservation analysis indicated that these variants were predominantly located in intolerant regions.

Segregation studies confirmed biallelic inheritance in every affected individual: in each compound-heterozygous case, the two variants were inherited in trans, as each parent carried only one of the two alleles, whereas unaffected relatives were either heterozygous carriers or non-carriers. The frameshift and one nonsense variant found in Family 3—c.456dup (p.Ala153Cysfs*27) and c.922G > T (p.Glu308*)—were novel and absent from control population databases. The c.922G > T variant had a CADD score of 46 and a BayesDel score of 0.66, and it was predicted to be pathogenic by multiple in silico tools. Because these variants introduced premature stop codons in an exon upstream of the final exon, nonsense-mediated mRNA decay was predicted, meeting the PVS1 very strong (PVS1_VS) criteria. According to the ACMG–AMP guidelines, both variants were classified as pathogenic. Detailed classifications of these variants are provided in Table 1 and Supplementary Table 1.

**Table 1:** Summary of in silico findings and ACMG classification for GAN variants, including variants not previously reported in other studies

Family	Family1	Family2	Family3	
Variant	c.808G > A, (pGly270Ser)	c.1478A > C, (p.Glu493Ala)	c.456dup, (p.Ala153Cysfs*27)	c.922G > T, (p.Glu308*)
In silico analysis				
SIFT4G	0 (Deleterious)	0 (Deleterious)	NA	NA
Polyphen2	0.543 (Possibly damaging)	0.971 (Probably damaging)	NA	NA
FATHMM-MKL	0.992 (Deleterious)	0.9956 (Deleterious)	NA	0.9822 (Deleterious)
MutationTaster	1 (Uncertain)	1 (Uncertain)	NA	1 (Uncertain)
CADD (raw score)	5.13	4.89	NA	10.29
CADD (PHRED)	28.7	27.3	NA	46
BayesDel noAF	0.1428	0.3571	NA	0.66
Allele frequency				
gnomAD	0	0	0	0
jMorp (60KJPN)	0	0	0	0
Previously reported variant	+ (this case only)	+ (this case only)	−	−

In silico data	PP3 supporting	PP3 moderate		
Population data	PM2 supporting	PM2 supporting	PM2 supporting	PM2 supporting
Segregation data	PP4 moderate		PP4 supporting	PP4 supporting
Other data	PP2 supporting	PP2 supporting	PVS1 very strong	PVS1 very strong
	PM3 moderate	PM3 moderate		
		PM5 moderate		
Classification of ACMG criteria	Likely pathogenic	Likely pathogenic	Pathogenic	Pathogenic

NA, not available

### Clinical features

The mean age at onset was 4.4 years (range, 1.5–8). When stratified based on phenotype, the mean age of onset was 2.3 years (range, 1.5–3) in patients with the classical phenotype and 5.8 years (4.5–8) in those with the CMT-like phenotype. A summary of their clinical manifestations and nerve conduction study (NCS) findings of the aforementioned five patients from four families is provided in Table 2.

**Table 2:** Summary of clinical, electrophysiological, and neuroimaging features in five patients with GAN

	Family1		Family2	Family3	Family4
	Patient1	Patient2	Patient3	Patient4	Patient5
Class	Mild	Mild	Mild	Classic	Classic
Onset Age/Sex	4.5y/M	8y/F	5y/M	3y/M	1.5y/F
Examination Age	11y	9y	72y	13y	20y
Clinical feature					
Initial symptom	Gait disturbance	Gait disturbance	Gait disturbance	Gait disturbance	Gait disturbance
Muscle weakness	Distal, lower > upper limb	Distal	Distal	Distal, lower > upper limb	Distal, lower > upper limb
Sensory disturbance	+	−	+	+	+
Ambulation status	Independent	Independent	Unable	Unable	Unable
Deep tendon reflex	Decreased	Normal	Decreased	Decreased	Decreased
Pyramidal sign	+	−	−	−	−
Cerebellar sign	−	−	−	NA	+
Intellectual disability	−	−	−	−	Borderline(IQ83)
Kinky or tightly hair	−	−	−	+	+
Scoliosis	NA	−	+	+	+
Respiratory dysfunction	−	−	+(CPAP at night)	+ (at night)	+ (CPAP at night)
Other symptom	Polydactyly, pes cavus, equinus, hammer toe, diarrhea	Arthrogryposis	Vocal cord paralysis, ileus, sleep apnea syndrome, bladder and rectal disturbance	Pes cavus, dysphagia, cyclic vomiting	High palate, macrocephaly, vertigo, dizziness, constipation, cutaneous cold, precocious puberty
NCS					
Median					
CMAP(mV)	3.9	/	N.E	4.2	0.84
MCV(m/s)	56.1	/	N.E	51.4	47.3
SNAP(μV)	2.8	/	N.E	N.E	/
SCV(m/s)	53.4	/	N.E	N.E	/
Ulnar					
CMAP(mV)	/	/	N.E	3	3.57
MCV(m/s)	/	/	N.E	53.7	56.5
SNAP(μV)	/	/	N.E	N.E	/
SCV(m/s)	/	/	N.E	N.E	/
Tibial					
CMAP(mV)	2.1	5.2	N.E	0.3	N.E
MCV(m/s)	50.8	50.4	N.E	37	N.E
Sural					
SNAP(μV)	0.5	4.1	N.E	N.E	/
SCV(m/s)	53.6	52.6	N.E	N.E	/
Head MRI	Normal	NA	Mild hyperintensities of periventricular white matter	T2 high lesion at periventricular, bilateral cerebella hemisphere. atrophy at brainstem	T2 high lesion at medulla oblongata, periaqueductal grey matter of the midbrain, splenium of corpus callosum

CMAP, compound muscle action potential; CPAP, continuous positive airway pressure; MCV, motor conduction velocity; MRI, magnetic resonance imaging; NA, not available; NCS, nerve conduction study; N.E, not evoked; SCV, sensory conduction velocity; SNAP, sensory nerve action potential

### Patient 1 in Family 1

Patients 1 and 2 were siblings in Family 1 born to nonconsanguineous parents. Patient 1, whose detailed clinical information was previously reported [27], was an 11-year-old boy. He had normal developmental milestones, including social smiling at 3 months, head control at 5 months, independent walking at 15 months, and first words at 16 months. He had polydactyly, which was surgically corrected at 1 year old. Cognitive assessment using the Wechsler Intelligence Scale for Children, Fourth Edition (WISC-IV) revealed normal intellectual ability.

Gait instability initially arose during early childhood, gradually progressing to frequent falls. At 10 years old, he required a cane for ambulation. Physical examination revealed pes cavus, hammer toes, and equinovarus deformity. His hair was normal. His gait was characterized by foot drop and a wide-based stance. Although the patellar reflexes were hyperactive and the Babinski reflex was positive, the Achilles tendon reflexes were absent. Sensory examination revealed preserved touch and pain sensation but mild impairment of vibratory and proprioceptive senses. No apparent signs of cerebellar ataxia were noted. NCS revealed normal motor and sensory nerve conduction velocities with reduced compound muscle action potential (CMAP) and sensory nerve action potential (SNAP) amplitudes. Brain and spinal MRI revealed no abnormalities, including the absence of brain atrophy or white matter lesions. Peripheral nerve biopsy identified fewer myelinated fibers, with enlarged axonal spheroids and onion bulb formation. Neurofilament accumulation was observed in giant axons, and Schwann cells surrounded myelinated fibers.

### Patient 2 in Family 1

Patient 2 was a 9-year-old girl. Gait disturbance and frequent falls were noted in this patient at approximately 8 years of age. Concurrently conducted NCS revealed lower-limb axonal neuropathy. Physical examination at 9 years of age revealed lower-distal limb muscle weakness, whereas muscle atrophy or sensory disturbance were absent. Although she had no hair abnormalities, scoliosis, or bony deformities, she had contractures in the joints. She had normal muscle tonus and tendon reflexes but no pyramidal tract signs or cerebellar ataxia findings. Moreover, she could walk without assistance. NCS revealed decreased CMAP amplitudes in the tibial nerve and decreased SNAP amplitudes in the sural nerve. Conversely, motor nerve conduction velocity in the tibial nerve and sensory nerve conduction velocity in the sural nerve were normal (Table 2).

### Patient 3 in Family 2

Patient 3, whose details were also reported previously in a Japanese journal [28], was a 72-year-old man who first noticed gait difficulties at 5 years of age. He required a cane by 13 years old and became wheelchair-dependent by 17 years of age. His muscle weakness and sensory impairment gradually progressed, leading to difficulty with fine hand movements by 36 years old. Bladder dysfunction emerged at 43 years of age, followed by intestinal motility impairment at 50 years. At 54 years of age, polysomnography revealed severe obstructive sleep apnea (apnea–hypopnea index = 58.8), and nocturnal continuous positive airway pressure (CPAP) therapy was initiated. By 61 years of age, the patient developed slurred speech, dysphonia, and vocal cord paralysis, and he underwent sigmoidectomy and colostomy for paralytic ileus at 62 years. Neurological examination revealed reduced or absent tendon reflexes with no pathological reflexes. Manual muscle testing (MMT) disclosed grade 5 upper-limb proximal strength and distal strength of grade 2 on the right and grade 0 on the left. Lower-limb proximal strength was graded 2–3 together with complete distal paralysis (grade 0). He exhibited distal hypoesthesia, dysesthesia, and bladder/rectal dysfunction. His hair was normal, and spinal X-rays revealed scoliosis. NCS detected no recordable motor or sensory nerve responses, and the patient’s serum creatine kinase level was elevated (1341 IU/L). Brain MRI revealed mild periventricular white matter lesions but no significant atrophy or cortical abnormalities.

### Patient 4 in Family 3

**Fig. 2:**
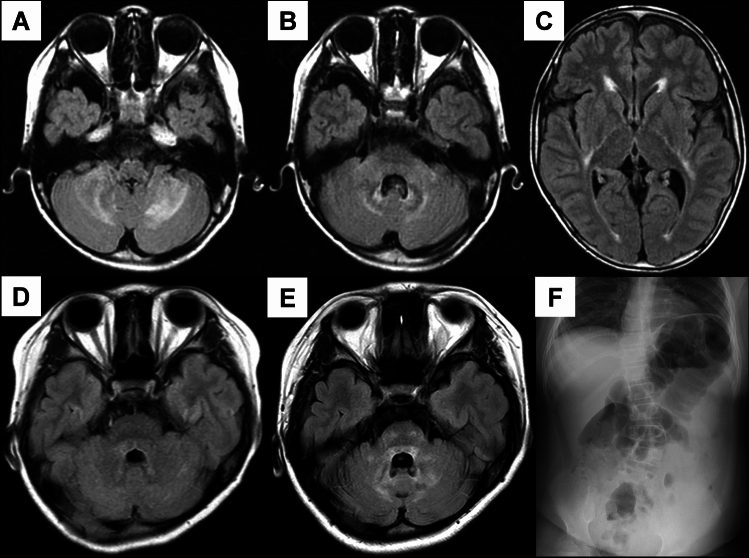
Brain MRI and spinal imaging of Patients 4 and 5 with classical GAN. **A**–**C** Axial FLAIR images of Patient 4 at 8 years old revealing high-intensity lesions in the periventricular white matter and bilateral cerebellar hemispheres, along with brainstem atrophy. **D**–**E** Axial FLAIR images of Patient 5 at 9 (**D**) and 14 years old (**E**). At 9, only a mild hyperintense lesion was observed in the periaqueductal gray matter of the midbrain. By 14 years of age, hyperintense lesions had expanded to the bilateral cerebellar hemispheres. **F** Abdominal radiograph of Patient 5 at 9 years old revealing scoliosis and bowel gas accumulation. FLAIR, fluid-attenuated inversion recovery

Patient 4, a 13-year-old boy, had been prone to falling since approximately 3 years of age. Additionally, muscle weakness was noted. Physical examinations revealed normal upper-limb muscle strength but predominant weakness and atrophy in the bilateral distal lower-limb muscles. Patellar and Achilles tendon reflexes were diminished, Babinski and Chaddock reflexes were negative, and no pyramidal tract signs were noted. Although he could initially walk unaided with steppage gait, he subsequently lost the ability to walk. Despite his ability to breathe spontaneously during the day, he requires a ventilator at night. Dysphagia was also identified; however, his intellectual ability was normal. Tight and curled hair (kinky hair-like) and scoliosis were noted. Electrophysiologic examination revealed sensorimotor axonal polyneuropathy with sensory nerve dominance. Head MRI disclosed periventricular white matter lesions and T2-weighted hyperintensities in the bilateral cerebellar hemispheres at 8 years of age (Fig. 2A–C). At 13 years of age, his white matter lesions were enlarged, and widespread cerebral atrophy was observed.

### Patient 5 in Family 4

Patient 5 was a 20-year-old woman born to nonconsanguineous parents. She could walk independently at 18 months old, but she exhibited early gait instability and frequent falls. By 6 years old, her dizziness and progressive gait disturbance worsened. At 8 years of age, she began luteinizing hormone-releasing hormone analog therapy for precocious puberty, and frequent falls (> 10/day) by 9 years old led to medical evaluation. Physical examination revealed thick hair, macrocephaly, a high-arched palate, a saddle nose, enlarged tonsils, scoliosis, and obesity (Fig. 2F). Although dizziness and gaze-evoked nystagmus were present, cerebellar ataxia was absent. Upper-limb strength was normal, whereas lower-limb strength was significantly reduced (MMT 3), affecting distal flexors and causing foot drop. She had a waddling and shuffling gait with no pyramidal or extrapyramidal signs. Hypoesthesia predominated in the lower extremities, and autonomic dysfunction manifested as constipation. WISC-IV assessment indicated borderline intellectual ability (IQ 83). Her visual acuity was normal. She started CPAP use at night at 10 years old. At 20 years old, her upper-limb strength remained normal, but severe lower-limb weakness necessitated wheelchair use. She exhibited mild dysarthria but maintained intact swallowing function. NCS identified sensorimotor axonal polyneuropathy with lower-limb predominance. Brain MRI revealed T2 hyperintensities in the medulla oblongata, periaqueductal region, and corpus callosum (Fig. 2D, E). Electroencephalogram showed spike-and-wave complexes despite no seizure history.

## Discussion

In this study, we conducted comprehensive genetic screening of 3315 Japanese patients with IPNs and identified five individuals from four unrelated families with *GAN* variants. All patients were born to nonconsanguineous parents. Three families harbored compound heterozygous variants, whereas one family carried a homozygous variant. Among the identified variants, one nonsense variant and one frameshift variant were novel, and both were classified as pathogenic according to the ACMG–AMP guidelines. Although all families had Japanese ancestry, they carried distinct variants, indicating that they originated from different lineages.

GAN is an extremely rare inherited neuropathy, with approximately 100 families reported globally [4, 29–31]. Because of its rarity, the population prevalence of GAN remains unclear [29]. In the current case series, the proportion of GAN among patients clinically suspected of having IPNs was 0.12% (4/3,315). Although most reported cases of GAN arose before 10 years of age [4, 30, 32], the proportion of patients with disease onset before 10 years of age was 0.46% (4/865) in this study. The reported prevalence of CMT varies widely, ranging from 3.1 to 82.3 per 100,000 individuals [33, 34]. A study in Japan estimated a prevalence of 10.8 per 100,000 [35], in line with the global prevalence of 17.69 per 100,000 reported in a meta-analysis [36]. This prevalence remains comparable even in early-onset CMT cases. Given that GAN accounted for 0.12% of all cases of CMT in this study, when extrapolated to the Japanese CMT prevalence, the estimated prevalence of GAN within the general population in Japan is approximately 0.013 per 100,000 individuals. However, this study might have underestimated the prevalence of GAN, as genetic screening for *GAN* mutations was not performed in patients with predominant CNS involvement. Further systematic screening, particularly in patients with early-onset neuropathy and unclassified neurodegenerative symptoms, is necessary to accurately determine the true population prevalence of GAN.

Of the four families, two exhibited the classical GAN phenotype, including peripheral neuropathy and additional systemic manifestations, whereas the other two families exhibited a phenotype primarily characterized by peripheral neuropathy, resembling CMT. Classical GAN is typically a severe disorder, often resulting in mortality within the second to third decades of life. Conversely, the CMT-like phenotype is generally typified by a more indolent disease course and more favorable life expectancy [7, 8]. In a large cohort of 45 patients, 10 (22.2%) exhibited a CMT-like phenotype with minimal or no CNS involvement [4]. In the same study, the mean age of onset for classical GAN and CMT-like phenotype were 2.3 and 5.4 years, respectively, concurrent with the older age of onset for the CMT-like phenotype in the present study. Following a similar trend, Patient 3 survived until 72 years old, which, to our knowledge, marks the longest survival of a patient with GAN in the English-language literature. Patient 3 did not exhibit hair abnormalities; however, he predominantly presented with lower-limb motor dysfunction, sensory impairment, and diminished deep tendon reflexes, consistent with a childhood-onset CMT phenotype. By 17 years of age, he required a wheelchair. Moreover, autonomic dysfunction emerged in adulthood, with bladder dysfunction, intestinal dysmotility, sleep apnea, and apparent CNS involvement presenting at 43, ~ 50, 54 years, and 61 years of age, respectively. The findings in this patient suggest that even in mild phenotypic presentations primarily characterized by peripheral neuropathy without overt systemic involvement, central and autonomic nervous system manifestations can develop with age. This observation supports the concept of a phenotypic continuum between classical GAN and its CMT-like variant, highlighting the need for long-term clinical surveillance in patients with atypical presentations.

The GAN gene does not have an obviously mutational hotspot, and pathogenic variants are widely distributed throughout the gene [4]. Missense, nonsense, frameshift, and splice site mutations and small insertions/deletions have all been reported as disease-causing variants [29]. No significant differences in disease severity or gigaxonin stability have been reported across different domains of the GAN gene [37]. However, GAN can arise from a loss-of-function mechanism, and previous reports indicated that biallelic null mutations, such as nonsense or frameshift variants, tend to be associated with severe phenotypes [4]. Moreover, recent studies suggested that disease severity can subtly vary depending on the affected domain. Specifically, variants in the Kelch domain have been associated with more severe phenotypes, followed by those in the broad-complex, tramtrack, and bric-à-brac (BTB) domain, whereas mutations in the BACK domain tend to result in relatively milder clinical presentations [5]. These findings suggest a potential, albeit limited, genotype–phenotype correlation, warranting further investigation. In this study, Patient 4 harbored one mutation in the BACK domain, but both variants were stop-gain mutations (nonsense or frameshift), leading to a classical GAN phenotype with disease onset at 1.5 years old. Conversely, Patient 5, who carried a missense variant in the Kelch domain, also presented with a severe phenotype, suggesting that the mutation type alone might not be sufficient to predict disease severity.

In patients with GAN, neurofilament aggregation in peripheral nerves leads to axonal swelling, resulting in thinly myelinated giant axons [1, 38]. NCS typically indicates an axonal neuropathy pattern, as previously reported [4, 8]. In this study, all patients who underwent NCS exhibited preserved conduction velocity consistent with axonal involvement. Additionally, kinky hair was observed in two families with the classical GAN phenotype and none from the patients with the CMT phenotype. Hair abnormalities in GAN are attributed to keratin dysfunction, an intermediate filament similar to neurofilament, and are considered a characteristic feature of GAN [38, 39]. Kinky hair is regarded as a clinical hallmark of classical GAN, but may occur less frequently in patients with milder or atypical phenotypes and therefore should not be considered as an exclusion criterion [40]. Moreover, cases of mild GAN without hair or CNS abnormalities primarily present with peripheral neuropathy, leading to a clinical diagnosis of CMT in some cases.

In 2024, a Phase 1 clinical trial evaluating an AAV vector-based gene therapy delivering a *GAN*-encoding transgene in 14 patients with GAN demonstrated promising therapeutic outcomes, including improvements in motor function, increased SNAP amplitudes, and enhanced neuroregeneration clusters in biopsy specimens [12]. However, the extreme rarity of GAN presents significant challenges in conducting double-blind, placebo-controlled trials, limiting the feasibility of large-scale clinical validation and delaying potential therapeutic implementation. It is imperative to enhance the identification and diagnosis of GAN to facilitate therapeutic advancements. Given the substantial phenotypic heterogeneity of GAN and its potential to present with atypical CMT-like phenotypes, cases lacking the hallmark clinical features might remain unrecognized or underdiagnosed. Notably, all four probands in this study were initially suspected of having CMT rather than GAN based on clinical presentation and NCS findings, leading to their inclusion in our IPN case series. Therefore, in patients with pediatric-onset CMT, even in the absence of CNS involvement or characteristic hair abnormalities, genetic screening for *GAN* should be systematically integrated to ensure accurate diagnosis and optimal clinical management.

This study had several limitations. Clinical information was obtained from multiple diagnostic institutions, and standardized scales were not uniformly applied to evaluate clinical findings. Although GAN exhibits a wide phenotypic spectrum, this study focused exclusively on Japanese patients, making it difficult to eliminate potential ethnic differences in disease presentation.

In conclusion, this comprehensive genetic screening of 3315 Japanese patients with clinically diagnosed IPNs identified seven *GAN* variants in five individuals from four unrelated families. Among them, two novel stop-gain mutations were classified as pathogenic according to the ACMG–AMP guidelines. Based on our findings, we estimated the prevalence of GAN to be 0.013 per 100,000 individuals. Furthermore, within the CMT-like phenotype, we identified a milder case with long-term survival beyond the conventional clinical spectrum of GAN. Owing to the phenotype heterogenicity of GAN, all cases in this study—classical or mild—were initially misdiagnosed as CMT. This study highlights the broad phenotypic variability of GAN, emphasizing the importance of comprehensive genetic analysis using next-generation sequencing in cases of pediatric-onset neuropathy to ensure the identification of *GAN* variants.

## Supplementary Information

Below is the link to the electronic supplementary material.

Supplementary file1 (DOCX 881 KB)

## Data Availability

Datasets are not readily available due to ethical and privacy restrictions. Requests should be directed to the corresponding author.
