# Supplementary material for: Charcot–Marie–Tooth-like presentation in giant axonal neuropathy: clinical variability and prevalence in a large Japanese case series
Source: J Neurol. 2025 Jul 16;272(8):514. doi: 10.1007/s00415-025-13243-5 (PMC12267338; doi:10.1007/s00415-025-13243-5)
Supplement: Supplementary file 1 — Supplementary file1 (DOCX 881 KB) [file 415_2025_13243_MOESM1_ESM.docx]

**Supplementary Table1. ACMG/AMP evidence-based classification and supporting data for GAN variants identified in this study**

(1) Abou Tayoun AN, Pesaran T, DiStefano MT, et al. Recommendations for interpreting the loss of function PVS1 ACMG/AMP variant criterion. Hum Mutat. 2018;39(11):1517-1524. doi:10.1002/humu.23626

(2) Karczewski KJ, Francioli LC, Tiao G, et al. The mutational constraint spectrum quantified from variation in 141,456 humans. Nature. 2020;581(7809):434-443. doi:10.1038/s41586-020-2308-7

(3) Tadaka S, Kawashima J, Hishinuma E, et al. jMorp: Japanese Multi-Omics Reference Panel update report 2023. Nucleic Acids Res. 2024;52(D1):D622-D632. doi:10.1093/nar/gkad978

(4) https://clinicalgenome.org/working-groups/sequence-variant-interpretation/

(5) Richards S, Aziz N, Bale S, et al. Standards and guidelines for the interpretation of sequence variants: A joint consensus recommendation of the American College of Medical Genetics and Genomics and the Association for Molecular Pathology. Genetics in Medicine. 2015;17(5):405-424. doi:10.1038/gim.2015.30

(6) Pejaver V, Byrne AB, Feng BJ, et al. Calibration of computational tools for missense variant pathogenicity classification and ClinGen recommendations for PP3/BP4 criteria. Am J Hum Genet. 2022;109(12):2163-2177. doi:10.1016/j.ajhg.2022.10.013

(7) Biesecker LG, Byrne AB, Harrison SM, et al. ClinGen guidance for use of the PP1/BS4 co-segregation and PP4 phenotype specificity criteria for sequence variant pathogenicity classification. Am J Hum Genet. 2024;111(1):24-38. doi:10.1016/j.ajhg.2023.11.009

**Supplementary Fig 1. Flowchart of genetic diagnosis**

**

**

In total, 3315 patients with IPNs, including 865 cases with disease onset before 10 years of age, were genetically analyzed. From 2007 to 2012, DNA microarray analysis targeted 28 IPN-related genes, excluding the GAN gene. Whole-exome sequencing was subsequently performed in 273 patients in whom no causative gene was identified, leading to the detection of biallelic *GAN* variants in two patients. Since 2012, genetic screening has been conducted using an in-house IPN-related gene panel, which includes the GAN gene, via next-generation sequencing. This approach identified two additional patients with biallelic *GAN* variants.
